# A new short version of the Cognitive Therapy Scale Revised (CTSR-4): preliminary psychometric evaluation

**DOI:** 10.1186/s40359-022-00730-x

**Published:** 2022-02-04

**Authors:** Sven Alfonsson, Georgios Karvelas, Johanna Linde, Maria Beckman

**Affiliations:** 1 Centre for Psychiatry Research, Department of Clinical Neuroscience, Karolinska Institutet, 171 77 Stockholm, Sweden; 2 Stockholm Health Care Services, Stockholm, Sweden

**Keywords:** CBT, Therapist competence, Psychotherapy training, Psychometrics

## Abstract

**Background:**

The value of using comprehensive but cumbersome coding instruments to assess therapeutic competency is unclear. Shorter, more general instruments may enable more research in this important area. The aim of this study was therefore to psychometrically evaluate a shorter version of the Cognitive Therapy Scale-Revised (CTSR) and to compare it with the full-length version.

**Methods:**

A four-item coding instrument (the CTSR-4) was derived from the CTSR. Four experienced psychotherapists used the CTSR-4 to assess 50 fifteen-minutes samples from audio-recorded CBT sessions. The criterion validity of the CTSR-4 was analyzed by comparing the results with previously expert-rated CTSR scores from the same sessions, and the inter-rater agreement between the three coders was calculated.

**Results:**

The CTSR-4 showed good criterion validity (ICC = .71–.88) when compared to the expert ratings of the complete CTSR, and the inter-rater agreement was adequate (ICC = .64–.79).

**Conclusions:**

A condensed version of the CTSR, used to assess CBT competence from shorter samples of therapy sessions, is moderately reliable and may provide similar results as the full-length version. According to preliminary analyses, the CTSR-4 has potential as a low-cost alternative to assess CBT competency in both research and psychotherapist training.

## Introduction

Over the years, much effort has been put into conceptualizing and defining psychotherapy competence in order to make it measurable [1, 2]. These efforts are also central to psychotherapist training, as it is assumed that competent therapists more efficiently treat patients [3]. Still, the relationship between therapists’ competence and clinical outcomes is far from clear [4, 5]. To provide more research in this area, adequate coding instruments are crucial. However, comprehensive and detailed measures of therapeutic competencies are resource-consuming, and the question is whether briefer and more general measures can provide satisfactory data to accurately assess therapeutic skills.

The Cognitive Therapy Scale (CTS) [6], or its revised version (CTSR), are the most used instruments in studies of CBT and its relation to patient outcome [7, 8]. However, the model of cognitive therapy (i.e., not empirical studies), and the ability to predict patient outcomes with the instruments is unclear [9]: Studies by Trepka et al. [10], Kazantzis et al. [11], Kuyken and Tsivrikos [12], and Liness et al. [13], found only weak associations between CBT competence and patient outcome, and Weck et al. [14], Bruijniks et al. [15] and Liness et al. [16] found no such associations. These ambiguous results highlight the question of how CBT competence should be measured, as well as the question of the impact of specific therapist skills on patient outcomes [17, 18].

A possible explanation for the inconclusive associations between CBT competence and patient outcome could be other mediating variables, such as the therapeutic alliance [19]. Another explanation could be that the associations are not linear—once a therapist competence level sufficient to help most patients, no further increase lead to improved patient outcome [13, 20, 21]. It is also possible that most patients’ needs are met by therapists of moderate competence, and that only a small group of patients require therapists with higher levels of competence [22–24]. These types of more detailed analyzes of CBT competence and patient outcomes are highly needed, but are hampered by the costs of coding instruments that analyze full sessions (e.g., CTSR). A more efficient alternative could be to analyze session samples. Weck et al. [25] assessed general competence rather accurately from either the start, middle, or end of CBT sessions. The specific skills, however, was more difficult to assess (e.g., homework could not be assessed from an initial part of a session). This problem could be minimized by selecting several shorter samples from a single session (e.g., start, middle and end).

Existing frameworks for psychotherapy competence propose a handful of general competencies, including theoretical knowledge, and the ability to engage patients, establish a working relationship, and transform theoretical knowledge into clinical practice [26, 27]. CBT also includes specific competencies, such as the structure of sessions and use of guided discovery. Competence measurements, such as the CTSR, typically include both general and specific competencies. Whether the effects of psychotherapy are best explained by specific- or general competencies is unknown [18, 28, 29], and no CBT study have yet found a specific competence highly associated with outcome. This may partly be explained by the fact that competence instruments, such as the CTSR, typically show high internal consistency, which questions the value of rating competence in numerous closely related subdomains [30]. A short instrument that rates therapists’ competence on a few selected global variables could be more efficient.

Briefer and more general instruments for CBT competence may both be more efficient and cost-effective. An important research question is therefore whether such less resource-consuming measures of therapy competence can show adequate psychometric properties. The aim of this study was to assess the criterion validity and inter-rater agreement of the CTSR-4, a brief instrument derived from the CTSR.

## Materials and methods

The criterion validity of the CTSR-4 was assessed by comparing scores from the CTSR-4 with previously independently assessed CTSR scores of the same CBT sessions. The inter-rater reliability of the CTSR-4 was assessed by comparing scores between independent coders for each session sample.

### The CTSR-4

**Fig. 1:**
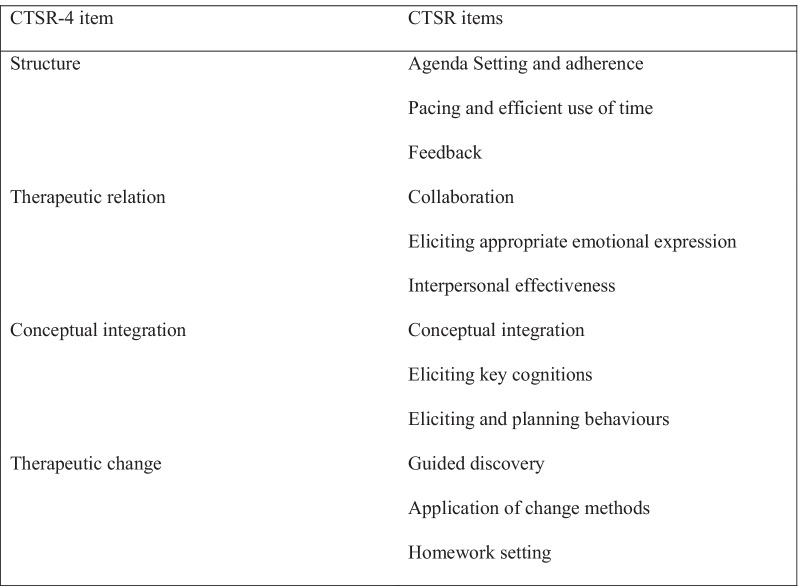
The items of the CTSR-4 and the corresponding CTSR items

The CTSR-4 was derived directly from the CTSR [7]. The CTSR comprises twelve items: Agenda Setting and adherence; Collaboration; Guided discovery; Feedback; Conceptual integration; Eliciting key cognitions; Eliciting appropriate emotional expression; Eliciting and planning behaviors; Application of change methods; Interpersonal effectiveness; Pacing and efficient use of time; and Homework setting. Although more research is needed, it is noteworthy that previous studies have suggested various factor structures of the CTSR without coming to a consensus and typically only the total score is calculated [9, 30, 31]. Each item is scored on a 7-point Likert scale from 0 to 6. A score of 0 indicates none or highly inadequate competence, 1–2 indicates inadequate competence, 3–4 indicates adequate competence, and a score of 5–6 indicates an expert level of competence in the corresponding domain. In the CTSR-4, the twelve CTSR items were merged into four items, each corresponding to three of the CTSR items; Structure, Therapeutic relation, Conceptual integration and Therapeutic change (Fig. 1). This four-factor structure was discussed and decided upon among the study authors and inspired by other researchers who have vented similar ideas [26]. The four items of the CTSR-4 arguably map well with the CBT competence framework [27]. Each item of the CTSR-4 is scored on a 7-point Likert scale from 0 to 6 and the total score can range from 0 to 24. A brief six-page scoring manual was developed, in which the CTSR-4 item descriptions and examples were provided to cover the broader scope of each item.

### Procedure

Four coders participated in this study. Two coders (A and B) assessed all 50 session samples while two coders (C and D) coded 20 and 30 session samples respectively. All coders had advanced level training in psychotherapy, had previously used the CTSR, and three of them were teaching university CBT courses and providing clinical supervision. The coders met for five to ten 90-min training sessions in different compositions, during which the CTSR-4 was discussed, and twelve audio recorded CBT sessions were coded independently and compared. Discrepancies in coding results were discussed to reach a consensus before the next training session. These recordings were only for training and were not used for further analyses. Over the training sessions, the inter-rater agreement increased among the coders and was eventually deemed adequate to independently code sessions. Inter-rater agreement could not be adequately statistically analyzed in the small training sample. Each coder received the manual and scoring sheets, and was instructed to listen to and independently assess the fifteen-minute session samples, and use approximately a total of 20 min for each assessment. The coders had additional meetings throughout the coding period to discuss the CTSR-4 items.

The 50 CBT sessions were randomly drawn from a set of 120 audio-recorded sessions, previously coded in full by independent expert raters using the CTSR. The expert raters were two CBT supervisors with more than fifteen years of clinical experience. They had had formal training in using the CTSR, and several years of experience in using the CTSR in psychotherapy training and supervision but were not part of the research team. The recorded CBT sessions were collected from six therapists working in open psychiatric care and included patients with different mood disorders and related problems. All therapists and patients had provided informed consent to participate in the study, and the study was approved by the Regional Ethics Committee Board (No. 2018/1735-31/3). The therapy sessions followed standard practice and comprised interventions and procedures common in mainstream CBT, such as Socratic dialogue, cognitive exercises, behavioral experiments, exposure exercises, problem-solving, and homework assignments. For this study, five minutes from the start (minutes 0–5) middle (typically minutes 22–27), and the end (typically minutes 45–50) were extracted for a total session sample of 15 min from each of the 50 CBT recordings. The recordings were blindly selected but edited so that only actual treatment time was included (e.g., recordings after the session had ended, where the therapist guided the patient back to the waiting room, was removed). Each edited 15-min recording was then given a random identifying number and provided to three of the four independent coders. Each recording was thus randomly distributed and coded by three of the four coders.

### Analysis

To be able to compare the results from the four items of the CTSR-4 with the full CTSR, the mean for the corresponding item scores from the CTSR was calculated. The scores were then rounded off to the nearest 0.5-value to facilitate statistical comparisons. To assess the criterion validity and inter-rater reliability of the CTSR-4, a two-way, random effects, absolute agreement model of Intra-Class Correlation (ICC) was used [32, 33]. For the criterion validity of the CTSR-4, the CTSR-4 and the full CTSR were compared, and for the CTSR-4 coders’ inter-rater agreement, the four coders’ ratings were compared. A single measure analysis was conducted for the validity analysis, while both single measure and average measure analyses were conducted for the inter-rater agreement analyses. An ICC < 0.50 was interpreted as poor, 0.50–0.75 as moderate, 0.75–0.90 as good and > 0.90 as excellent reliability [34]. According to published guidelines for calculating statistical power for inter-rater agreement, 50 observations coded by four raters should obtain a power of at least 90% to identify weak associations (ICC > 0.3) between ratings [35].

## Results

The CTSR-4’s and the CTSR’s mean values are summarized in Table 1. Overall, the CTSR-4 coders’ scores were distributed evenly around the CTSR raters’ mean scores with only small deviations in means and standard deviations. For most items, the CTSR-4 coders’ mean scores were within 0.1 point of the expert raters’ scores. The one exception was coder D’s mean score on Concept which was somewhat lower than the other coders’ mean scores and the expert raters’ mean score.

**Table 1:** Expert rater and coder mean values, standard deviations and range for each CTSR-4 item

CTSR-4 item	CTSR expert raters m (SD), range	Coder A m (SD), range	Coder B m (SD), range	Coder C m (SD), range	Coder D m (SD), range
Structure	3.2 (0.70), 1.0–4.5	3.3 (0.78), 1.0–5.0	3.2 (0.98), 1.0–4.5	3.5 (0.81), 1.5–5.0	3.4 (0.84), 1.5–5.0
Relation	3.5 (0.86), 1.0–5.0	3.5 (0.76), 1.0–5.0	3.4 (0.98), 2.0–5.0	3.5 (0.80), 2.0–5.0	3.4 (0.76), 2.0–5.0
Concept	3.2 (0.95), 0.5–5.0	3.3 (1.00), 1.0–4.5	3.4 (1.14), 1.0–5.0	3.2 (1.12), 1.0–5.0	2.9 (1.14), 1.0–5.0
Change	3.4 (0.95), 1.0–5.0	3.5 (1.01), 1.0–5.0	3.4 (1.14), 1.0–5.0	3.5 (0.87), 1.0–5.0	3.4 (0.72), 2.0–5.0
Total score	13.3 (3.00), 7.0–18.5	13.5 (3.07), 5.0–19.5	13.4 (3.56), 6.0–19.0	13.7 (3.13), 7.0–19.0	13.0 (2.89), 7.0–18.5

CTSR, Cognitive Therapy Scale Revised

### Analysis of criterion validity

The ICC for the CTSR expert raters and each CTSR-4 coder ranged from 0.71 to 0.88 (Table 2) for the individual coders, which corresponds to moderate to good levels of agreement and from 0.89 to 0.96 for the coder mean which corresponds to good to excellent agreement [34].

**Table 2:** The criterion validity (ICC) comparing the expert rating with each CTSR-4 coder and the coder mean

CTSR-4 item	Coder A ICC (95% CI)	Coder B ICC (95% CI)	Coder C ICC (95% CI)	Coder D ICC (95% CI)	Coder mean ICC (95% CI)
Structure	.86 (.77–.92)	.74 (.59–.85)	.75 (.42–.88)	.71 (.24–.88)	.91 (.82–.96)
Relation	.85 (.75–.91)	.71 (.54–.82)	.81 (.69–.89)	.75 (.54–.87)	.89 (.81–.94)
Concept	.88 (.80–.93)	.79 (.64–.88)	.83 (.72–.90)	.82 (.66–.91)	.90 (.83–.95)
Change	.86 (.77–.92)	.75 (.60–.85)	.80 (.67–.88)	.75 (.53–.87)	.90 (.83–.94)
Total score	.93 (.88–.96)	.83 (.72–.90)	.86 (.76–.92)	.83 (.67–.91)	.96 (.92–.98)

### Inter-rater agreement

The inter-rater agreement for the four coders ranged from 0.64 (Structure and Relation) to 0.79 (Concept) in the single measure analysis, and between 0.84 (Structure and Relation) to 0.92 (Concept) in the average measure analysis (Table 3). This corresponds to moderate and good inter-rater reliability, respectively [34]. Thus in both analyses, Structure and Relation had the lowest inter-rater reliability and Conceptualization had the highest level of agreement.

**Table 3:** Inter-rater agreement (ICC) between the four CTSR-4 coders

CTSR-4 item	Single measure ICC (95% CI)	Average measure ICC (95% CI)
Structure	.64 (.49–.76)	.84 (.74–.91)
Relation	.64 (.50–.76)	.84 (.75–.90)
Concept	.79 (.69–.87)	.92 (.87–.95)
Change	.69 (.56–.80)	.87 (.79–.92)
Total	.76 (.64–.84)	.90 (.84–.94)

## Discussion

The aims of this study were to assess the criterion validity and inter-rater agreement of the CTSR-4 when evaluating short samples of CBT sessions. The validity was assessed by comparing the results from four CTSR-4 coders with previously expert-rated CTSR scores. The results showed ICC-values between 0.71 and 0.88, indicating a good level of agreement [7, 11]. While there were some minor differences between the coders, there were no systematic deviations, and the scores of the four coders were distributed evenly around the expert rater’s values. The variance across items was low for all four coders, indicating that none of the specific CTSR-4 items were easier or more difficult to assess. Overall, the results showed a satisfactory criterion validity of the CTSR-4.

The inter-rater reliability results also indicated moderate to good agreement for the single measures, and good to excellent agreement for the average measures [32]. Again, these results are on par or slightly higher than those found for the CTSR in previous studies [7, 36], which is expected when collapsing an instrument into fewer items. Taken together, the results from the current analyses show that a shorter measure of competence, such as the CTSR-4, can present adequate psychometric properties when coding samples of CBT sessions. That the inter-rater reliability ICC-values were somewhat lower than the criterion validity ICC-values indicates that the CTSR-4 coders’ scores were distributed both above and below the expert raters’ scores so that they were actually in more agreement to the expert raters than to each other.

The CTSR-4 was created by merging the original twelve items of the CTSR into four, generating a more global measure of CBT competence. Previous studies indicate high to very high internal consistency of the CTSR (i.e., the original study of the CTSR found alpha values of 0.92 to 0.97 [7]. In other words, there is a high probability that, for any given CBT session, items on the CTSR will receive the same score. Whether this is an effect of the instrument or the coding, or whether this is inherent in the concept of CBT competence, is unknown. The CTSR-4 investigated in this study was designed based on a theoretical understanding of CBT while recent more data-driven studies have suggested that the CTSR comprise one to three distinct factors [9, 30]. In either case, merging the CTSR items into fewer variables may be efficient when assessing general CBT competence. However, four broader items may make the CTSR-4 less sensitive to variance in CBT competencies. A therapist may, for example, show high competence in guided discovery, and at the same time no, or little, competence in setting up homework assignments. Such variances in CBT competencies and skills may be easier to detect with the CTSR than the CTSR-4, in which these two competencies are included within the same item. The CTSR may therefore be more useful when the goal is to map therapist competence and identify areas for further development. However, no study has yet shown that CTSR can identify such variances in CBT competence, and the results of previous studies arguably point in the direction that a competent therapist shows similar levels of competence across therapeutic domains [30, 37]. Also, while most agree that some CBT components, such as setting the agenda and eliciting key cognitions, are hallmarks of quality CBT, there is very little research confirming that specific CBT components are essential for patient outcomes (however, see [38, 39]. Recent research has also shown that sometimes skills specific to a treatment manual or model may be more important than general CBT skills for treatment outcomes [15, 40]. Still, the CTSR, and the theoretical model of CBT competences that it relies on, remain highly important as therapist training tools, since they describe therapist competence in higher detail than the CTSR-4, and therefore more clearly present the full spectra of therapist behaviors.

A limitation of this study was the small number of coders, and the restricted number of sessions assessed. The four coders had advanced level training in CBT, and long experience in clinical work and supervising CBT therapists. It is still unclear to what degree their experiences and training may have affected their assessments, and to what degree the results can be generalized to coders with lower levels of experience and training [41]. Similarly, the fifty sessions of CBT that were assessed may have been too homogenous and not representative of CBT sessions generally. The range of the scores in both the CTSR-4 and the CTSR items were somewhat restricted with no item receiving a score of six. To conduct rigorous psychometric testing, a larger and more varied sample of sessions may be needed. However, based on published guidelines, the study was designed to have enough power to detect low degrees of associations between variables, and all of the associations found were statistically significant. A further limitation was the informal training of the coders in using the CTSR-4. All four had experience of using the CTSR, but no formal training in using the instrument. Instead, a series of workshops were arranged, in which the coders discussed their ratings to reach consensus. Additional workshops with joint coding may have resulted in higher levels of inter-rater agreement among the coders, though this needs to be confirmed in future studies. Lastly, in this study, the association between the CTSR-4 scores and patient outcomes could not be analyzed. It is important to remember that, in the end, therapeutic competence should be decided upon the therapeutic skills and behaviors necessary to benefit patients [42].

Taken together, this study shows that a new shorter instrument, such as the CTSR-4, derived from the CTSR, can provide satisfactory criterion validity and inter-rater reliability when evaluating samples of CBT sessions. The correlations between the coders’ and the expert raters’ scores were all in the good range, indicating that the CTSR-4 can be used to efficiently rate CBT competence in a similar way as CTSR. Since the CTSR-4 comprise only four items, and can be used to analyze 15-min samples of CBT sessions, the instrument may facilitate further research of CBT competence and therapist training. Future studies need to confirm the conclusions in larger samples as well as investigate possible associations between CBT competence and patient outcomes.

## Data Availability

The datasets analyzed during the current study are available from the corresponding author on reasonable request.
